# Extent and prognostic value of MGMT promotor methylation in glioma WHO grade II

**DOI:** 10.1038/s41598-020-76312-x

**Published:** 2020-11-12

**Authors:** Philipp Karschnia, Nico Teske, Mario M. Dorostkar, Sebastian Siller, Jonathan Weller, Joachim M. Baehring, Jorg Dietrich, Louisa von Baumgarten, Jochen Herms, Joerg-Christian Tonn, Niklas Thon

**Affiliations:** 1 Division of Neuro-Oncology, Department of Neurosurgery, Ludwig Maximilians University School of Medicine, Marchioninistrasse 15, 81377 Munich, Germany; 2 German Cancer Consortium (DKTK), Partner Site, Munich, Germany; 3 Division of Neuro-Oncology, Department of Neurology, Yale School of Medicine, New Haven, CT USA; 4 Division of Neuro-Oncology, Department of Neurology, Massachusetts General Hospital Cancer Center, Harvard Medical School, Boston, MA USA; 5 Center for Neuropathology and Prion Research, Ludwig-Maximilians-University School of Medicine, Munich, Germany

**Keywords:** CNS cancer, Biomarkers, Risk factors, DNA methylation, Surgical oncology, Neurological disorders

## Abstract

MGMT promotor methylation is associated with favourable outcome in high-grade glioma. In glioma WHO grade II, it is unclear whether the extent of MGMT promotor methylation and its prognostic role is independent from other molecular markers. We performed a retrospective analysis of 155 patients with glioma WHO grade II. First, all 155 patients were assigned to three molecular groups according to the 2016 WHO classification system: (1) oligodendroglioma, IDH-mutant and 1p19q co-deleted (n = 81); (2) astrocytoma, IDH-mutant and 1p19q non-codeleted (n = 54); (3) astrocytoma, IDH-wildtype (n = 20). MGMT promotor methylation was quantified using Sanger sequencing of the CpG sites 74–98 within the MGMT promotor region. Highest numbers of methylated CpG sites were found for oligodendroglioma, IDH-mutant and 1p19q co-deleted. When 1p19q co-deletion was absent, numbers of methylated CpG sites were higher in the presence of IDH-mutation. Accordingly, lowest numbers were seen in the IDH-wildtype subpopulation. In the entire cohort, larger numbers of methylated CpG sites were associated with favourable outcome. When analysed separately for the three WHO subgroups, a similar association was only retained in astrocytoma, IDH-wildtype. Collectively, extent of MGMT promotor methylation was strongly associated with other molecular markers and added prognostic information in astrocytoma, IDH-wildtype. Evaluation in prospective cohorts is warranted.

## Introduction

WHO grade II gliomas represent a heterogeneous group of primary central nervous system neoplasms arising from the supporting glial cells of the cerebral parenchyma. Molecular markers rather than histological classification alone have been found to be prognostically relevant for outcome in such tumors. Accordingly, in 2016 the World Health Organization (WHO) has introduced molecular groups based on the presence of isocitrate dehydrogenase 1/2 (IDH) mutation and 1p19q co-deletion into the classification of glioma WHO grade II^1^. Since then, the overwhelming impact of molecular markers has further been revealed as summarized by the latest cIMPACT-NOW update^2^. This particularly concerns gliomas without IDH mutations which show a less favourable prognosis in the presence of an additional TERT mutation, EGFR amplification, or aberration on chromosomes 7 and 10.

Methylation of the promotor region of the O6-methylguanine-DNA-methyltransferase (MGMT) gene is another molecular marker which is associated with more favorable outcome in glioma WHO grade III and IV^3,4^. It is less well-defined whether MGMT promotor status also adds prognostic information in glioma WHO grade II. Although such an association has been suggested in previous studies, it is unclear whether the extent and prognostic effect of MGMT promotor methylation are independent from the presence of IDH mutation or 1p19q co-deletion^5,6^.

In the present study, we describe a large cohort of 155 adult patients with histologically verified glioma grade II according to the WHO 2016 classification treated at a single academic neuro-oncology centre. Based on this cohort, we outline the extent of MGMT promotor methylation and its potential prognostic value in patients with glioma WHO grade II.

## Materials and methods

### Study population

We searched the institutional database of the Center for Neuro-Oncology at the Ludwig Maximilians University School of Medicine for adult patients with histologically verified supratentorial glioma WHO grade II seen between 2015 and 2019 in our interdisciplinary brain tumor board. Histopathological diagnosis was based upon tissue sampled during microsurgical tumor removal, or stereotactic biopsies in lesions of uncertain differential diagnosis or where safe resection appeared not feasible. Tumors were retrospectively classified according to the 2016 revised WHO classification system^1^. Accordingly, patients were assigned to one of three molecular groups of glioma WHO grade II: (1) oligodendroglioma, IDH mutant and 1p19q co-deleted; (2) astrocytoma, IDH mutant and 1p19q non-codeleted; (3) astrocytoma, IDH wild-type. Patients in which 1p19q, IDH, or MGMT promotor status were unavailable for review were excluded from the study.

Diagnostic and treatment decisions were based upon interdisciplinary brain tumor board recommendations and patient preference. Institutional guidelines with follow-up imaging every six months or in case of any clinical deterioration were followed. In patients with radiographically suspected malignant progression, ^18^F-FET-PET and imaging-guided minimal-invasive stereotactic biopsy in case of remaining uncertainty were provided^7,8^. We collected demographic and clinical information, histopathology, molecular markers, radiographic and other diagnostic findings, treatment specifics, and clinical outcome. Database closure was September 1, 2019. Study design and methods were approved by the Institutional Review Board of the Ludwig Maximilians University in Munich, Germany (AZ 20-063) with a waiver of informed consent. The study protocol conformed to the ethical guidelines of the 1975 Declaration of Helsinki, as revised in 1983. All data were kept anonymous.

### MGMT promotor methylation and molecular markers

MGMT promotor status was analysed using Sanger sequencing of the Cytosine-Guanine dinucleotide (CpG) sites 74–98 within the MGMT promotor region as previously described^9,10^. Methylation of each individual CpG site was defined as ratio of cytosine/thymine peak > 50%. The total number of methylated CpG sites was calculated for each patient. Raters were blinded for clinical outcome data. In addition, tumor tissue was frequently assessed for expression of alpha-thalassemia/mental retardation syndrome X-linked protein (ATRX) per immunohistochemistry and telomerase reverse transcriptase promotor (TERT) mutations per Sanger sequencing.

### Statistical analysis

Statistical analysis was performed as previously described^11,12^. The D' Agostino-Pearson omnibus normality test was used to test for normal distribution of numerical data. Differences between two parametric groups were tested by the Student’s t test, differences between two non-parametric groups by the Mann–Whitney U-test, and differences among more than two groups by ANOVA. If not indicated otherwise, values are expressed as mean ± standard error of the mean. Categorical variables are given in absolute numbers and percentage. Relationships between two or more categorical variables were analysed using the chi-square test. For survival analyses, patients were followed until death or day of database closure (September 1, 2019). Patients lost to follow-up were censored at day of last follow-up. Date of diagnosis was set as date of pathological glioma WHO grade II confirmation. Date of radiographic progression was defined as date when diagnosis of radiographic progression resulting in a therapeutic consequence was made, or death from disease-related causes. Date of malignant progression was defined as date when tissue-based diagnosis of malignant progression to WHO grade III or IV was made, or death from disease-related causes. Overall survival was defined as interval from diagnosis to death from any cause. Follow-up, survival, and predictors of outcome were calculated using Kaplan–Meier survival analysis and log-rank test. Statistical analyses were performed using Prism statistical software (Prism 7.0a; GraphPad Software Inc., San Diego, CA, USA). The significance level was set at *p* ≤ 0.05.

## Results

### Study population

Overall, we identified a total of 182 patients with supratentorial glioma WHO grade II. 27 patients were excluded from the present study because 1p19q, IDH, or MGMT promotor status were unavailable for review. 155 patients with supratentorial glioma WHO grade II were therefore included in the present study (Table 1). Based on histopathology, 81 oligodendrogliomas (81/155 patients, 52%), 68 diffuse astrocytomas (44%), 3 gemistocytic astrocytomas (2%), 2 pleomorphic xanthoastrocytomas (1%), and 1 protoplasmic astrocytoma (1%) were encountered. Based on molecular signature, we retrospectively assigned the study population to one of the following three groups: (1) oligodendroglioma, IDH mutant and 1p19q co-deleted (81/155 patients, 52%); (2) astrocytoma, IDH mutant and 1p19q non-codeleted (54/155, 35%); and (3) astrocytoma, IDH wild-type and 1p19q non-codeleted (20/155, 13%).

**Table 1:** Patient characteristics for glioma WHO grade II assigned according to the 2016 WHO classification.

Molecular markers	1p19q codel IDHmut	1p19q non-codel IDHmut	1p19q non-codel IDHwt	Total	*p*-value
**Overall, n (%)**	81	54	20	155	
**Histopathology**					
ODG	81 (100%)	0	0	81 (52%)	
Diffuse AST	0	50 (93%)	18 (90%)	68 (44%)	
Gemistocytic AST	0	3 (6%)	0	3 (2%)	
PXA	0	0	2 (10%)	2 (1%)	
PP AST	0	1 (2%)	0	1 (1%)	
**Age, years**					
18–35	26 (32%)	28 (52%)	3 (15%)	57 (37%)	*0.001
36–50	37 (46%)	21 (39%)	4 (20%)	62 (40%)	
51–65	14 (17%)	5 (9%)	5 (25%)	24 (16%)	
> 65	4 (5%)	0	8 (40%)	12 (8%)	
**Gender**					
Female	45 (56%)	24 (44%)	10 (50%)	79 (51%)	0.447
Male	36 (44%)	30 (56%)	10 (50%)	76 (49%)	
**KPS**					
< 90	9 (11%)	3 (6%)	6 (30%)	18 (12%)	0.074
90–100	62 (77%)	44 (82%)	12 (60%)	118 (76%)	
n.a	10 (12%)	7 (13%)	2 (10%)	19 (12%)	
**Tumor diameter**					
0–2.5 cm	8 (10%)	8 (15%)	4 (20%)	20 (13%)	0.195
2.6–5 cm	33 (41%)	23 (43%)	8 (40%)	64 (41%)	
5.1–7.5 cm	24 (30%)	20 (37%)	6 (30%)	50 (32%)	
> 7.5 cm	14 (17%)	3 (6%)	2 (10%)	19 (12%)	
n.a	2 (3%)	0	0	2 (1%)	
**Methylated CpG sites**					
0–8	0	3 (6%)	8 (40%)	11 (7%)	*0.001
9–16	19 (24%)	21 (39%)	4 (20%)	44 (28%)	
17–25	62 (77%)	30 (56%)	8 (40%)	100 (65%)	
**Molecular markers**					
ATRX wild-type	65 (80%)	5 (9%)	17 (85%)	87 (56%)	*0.001
ATRX loss	1 (1%)	44 (82%)	1 (5%)	46 (30%)	
ATRX status n.a	15 (19%)	5 (9%)	2 (10%)	22 (14%)	
TERT wild-type	3 (4%)	32 (59%)	3 (15%)	38 (25%)	*0.001
TERT mutation	41 (51%)	3 (6%)	14 (70%)	58 (37%)	
TERT status n.a	37 (46%)	19 (35%)	3 (15%)	59 (38%)	
**First-line therapy**					
*Surgical resection*					0.495
GTR	10 (12%)	9 (17%)	2 (10%)	21 (14%)	
STR	8 (10%)	6 (11%)	0	14 (9%)	
*Chemotherapy*					*0.007
TMZ	16 (20%)	8 (15%)	3 (15%)	27 (17%)	
PCV	1 (1%)	0	0	1 (1%)	
PC	14 (17%)	2 (4%)	0	16 (10%)	
TMZ + PC	3 (4%)	1 (2%)	0	4 (3%)	
Radiotherapy	2 (3%)	6 (11%)	2 (10%)	10 (7%)	0.106
Radiochemotherapy	12 (15%)	2 (4%)	5 (25%)	19 (12%)	*0.028
Brachytherapy	4 (5%)	8 (15%)	0	12 (8%)	*0.042
Wait-and-scan	18 (22%)	18 (33%)	4 (20%)	40 (26%)	0.287

Characteristics are given for glioma WHO grade II patients with 1p19q co-deletion, IDH mutant (n = 81); without 1p19q co-deletion, IDH mutant (n = 54); without 1p19q co-deletion, IDH wild-type (n = 20); and are summarized for all patients (total; n = 155). *P*-values are given for numerical and dichotomous variables. Asterisks indicate *p* ≤ 0.05.

*AST* astrocytoma, *ATRX* alpha-thalassemia/mental retardation syndrome X-linked protein, *CpG* cytosine-guanine dinucleotide, *GTR* gross total resection, *IDHmut* isocitrate dehydrogenase 1/2 mutation, *IDHwt* isocitrate dehydrogenase 1/2 wild-type, *KPS* Karnofsky performance score, *MGMT* O6-methylguanine-DNA methyltransferase promotor, *n.a.* not available for review, *ODG* oligodendroglioma, *PC* procarbazine, lomustine, *PCV* procarbazine, lomustine, vincristine, *PP* protoplasmic, *PXA* pleomorphic xanthoastrocytoma, *STR* subtotal resection, *TERT* telomerase reverse transcriptase promotor, *TMZ* temozolomide, *1p19q codel* 1p19q co-deleted, *1p19q non-codel* 1p19q non-codeleted.

### Demographic and clinical findings

Among the three molecular groups, median age at diagnosis was highest for patients with astrocytoma, IDH wild-type (56 ± 3.9 years; range 19–81) followed by patients with oligodendroglioma, 1p19q co-deleted (43 ± 1.4 years; range 20–73) and those with astrocytoma, IDH mutant (37 ± 1.3 years; range 21–62) (*p* = 0.001). Male-to female ratio and Karnofsky performance score did not differ between the three molecular groups (male-to-female-ratio: *p* = 0.447; Karnofsky performance score: *p* = 0.074).

**Figure 1:**
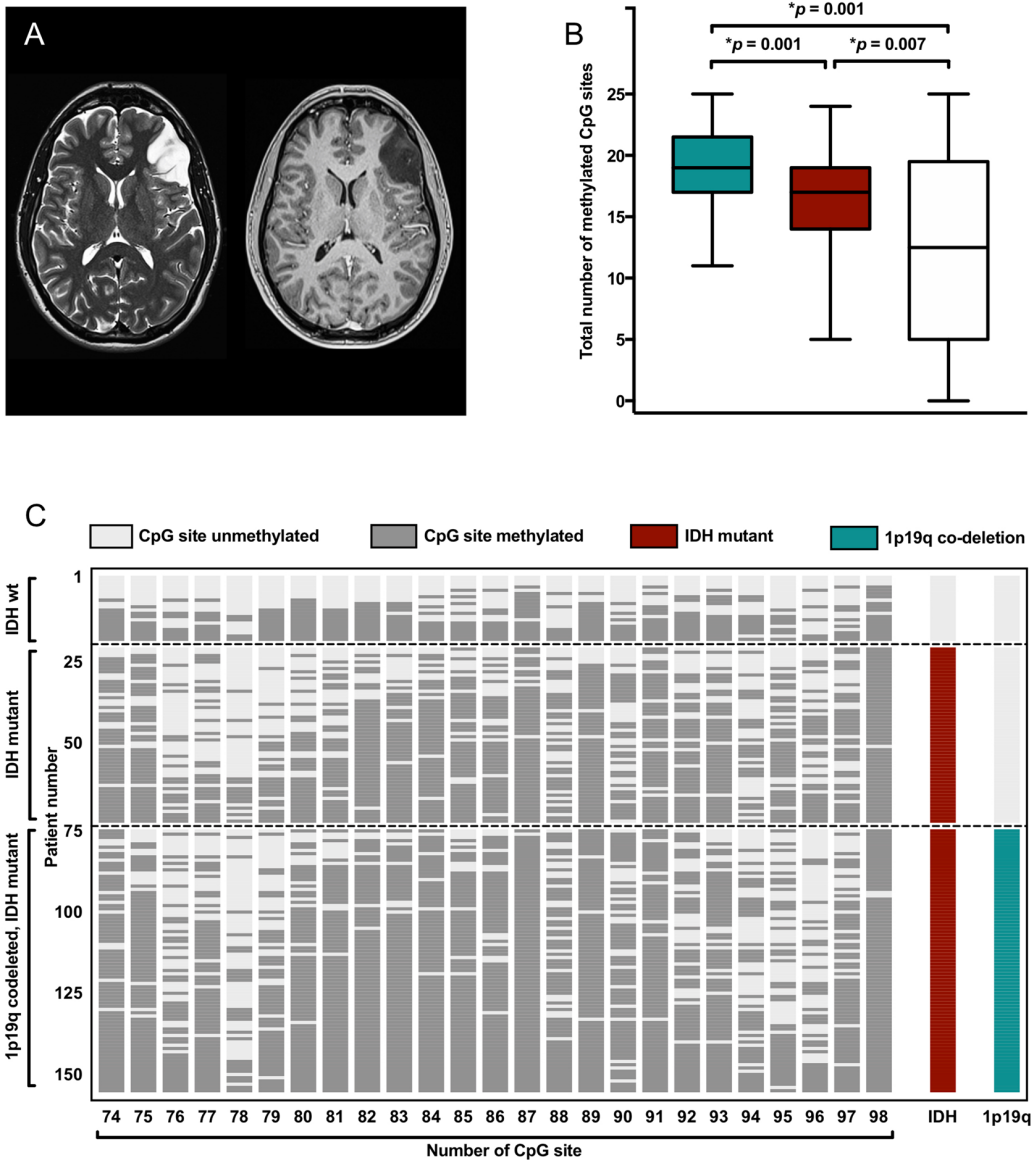
Extent and pattern of MGMT promotor methylation. (**A**): Axial brain MRI with T2-weighted (left panel) and T1-weighted post-contrast (right panel) sequences shows diffuse astrocytoma without contrast-enhancement. (**B**) Number of methylated CpG sites in patients with 1p19q co-deleted oligodendroglioma (cyan), IDH mutant astrocytoma (red), and IDH wild-type astrocytoma (white). Median, interquartile range, and total range are given. (**C**) Methylation pattern of CpG sites 74–98 within the MGMT promotor region in 155 patients with glioma WHO grade II. Each row corresponds to an individual patient, and each column to a different CpG site. Dark grey rectangles represent methylated sites and light grey rectangles represent unmethylated sites. IDH and 1p19q status are also indicated for each patients and color-coded.

Pre-treatment brain magnetic resonance imaging frequently demonstrated hyperintense lesions with ill-defined margins on T2-weighted sequences (Fig. 1A). Tumors were most often located in the frontal lobe, less often in the temporal or insular lobes, and only in selected cases in the parietal or occipital lobes. There was no clear difference in regard of tumor localization between the three molecular groups. Maximal tumor diameter was measured on axial T2-weighted sequences, and there was no difference between individuals with oligodendroglioma, 1p19q co-deleted and astrocytoma, IDH mutant or astrocytoma, IDH wild-type (5.2 ± 0.3 cm vs. 4.7 ± 0.3 cm vs. 4.5 ± 0.4 cm, respectively; *p* = 0.195). Generalised or focal seizures were the most commonly reported symptoms (87/155 patients, 56%). Seizures were most frequently encountered in patients with temporal or frontal tumor localization, and did not differ in semiology and frequency between the three molecular groups (oligodendroglioma, 1p19q co-deleted: 47/81 patients, 58%; astrocytoma, IDH mutant: 29/54 patients, 54%; astrocytoma, IDH wild-type: 11/20 patients, 55%; *p* = 0.879).

### Extent of MGMT promotor methylation

In the entire cohort, the mean number of methylated CpG sites was 17.1 ± 0.4 (68.4 ± 1.6% of 25 CpG sites) and did strongly vary between individual patients (range 0–25) (Fig. 1B). The mean number of methylated CpG sites was 18.9 ± 0.4 (75.6 ± 1.6%; range 11–25) among patients with oligodendroglioma, 1p19q co-deleted and was found to be higher when compared to 16.3 ± 0.6 methylated CpG sites (65.2 ± 2.4%; range 5–24) in patients with astrocytoma, IDH mutant (*p* = 0.001). In turn, the number of methylated CpG sites in patients with astrocytoma, IDH mutant was higher when compared to 12.3 ± 1.9 methylated CpG sites (49.2 ± 7.6%; range 0–25) in patients with astrocytoma, IDH wild-type (*p* = 0.007). This finding was also true when the patients with pleomorphic xanthastrocytomas were excluded from the analysis. Also, there was greater range in the individual number of methylated CpG sites among patients with astrocytoma, IDH wild-type when compared to the two other molecular groups. Of note, in all three groups some CpG sites such as CpG site number 87 and 98 were more frequently found to be methylated than others (Fig. 1C).

### Treatment and outcome

First-line management of glioma WHO grade II included microsurgical tumor resection, chemotherapy (predominantly temozolomide or procarbazine/lomustine), involved-field radiotherapy, combined radiochemotherapy, interstitial brachytherapy, and wait-and-scan approaches (Table 1). The number of patients which have received surgical tumor resection, radiotherapy, or wait-and-scan approaches was comparable between the three molecular groups. Patients allocated to first-line chemotherapy predominantly had oligodendroglioma, 1p19q co-deleted. Radiochemotherapy was most often provided in astrocytoma, IDH wild-type. Of note, we only encountered patients with ≥ 11 methylated CpG sites which have received chemotherapy or radiochemotherapy. Treatment approaches administered after tumor progression included microsurgical tumor resection, chemotherapy, radiotherapy, and interstitial brachytherapy. No clear difference in regard of salvage therapy after tumor progression was noted between the three molecular groups.

**Figure 2:**
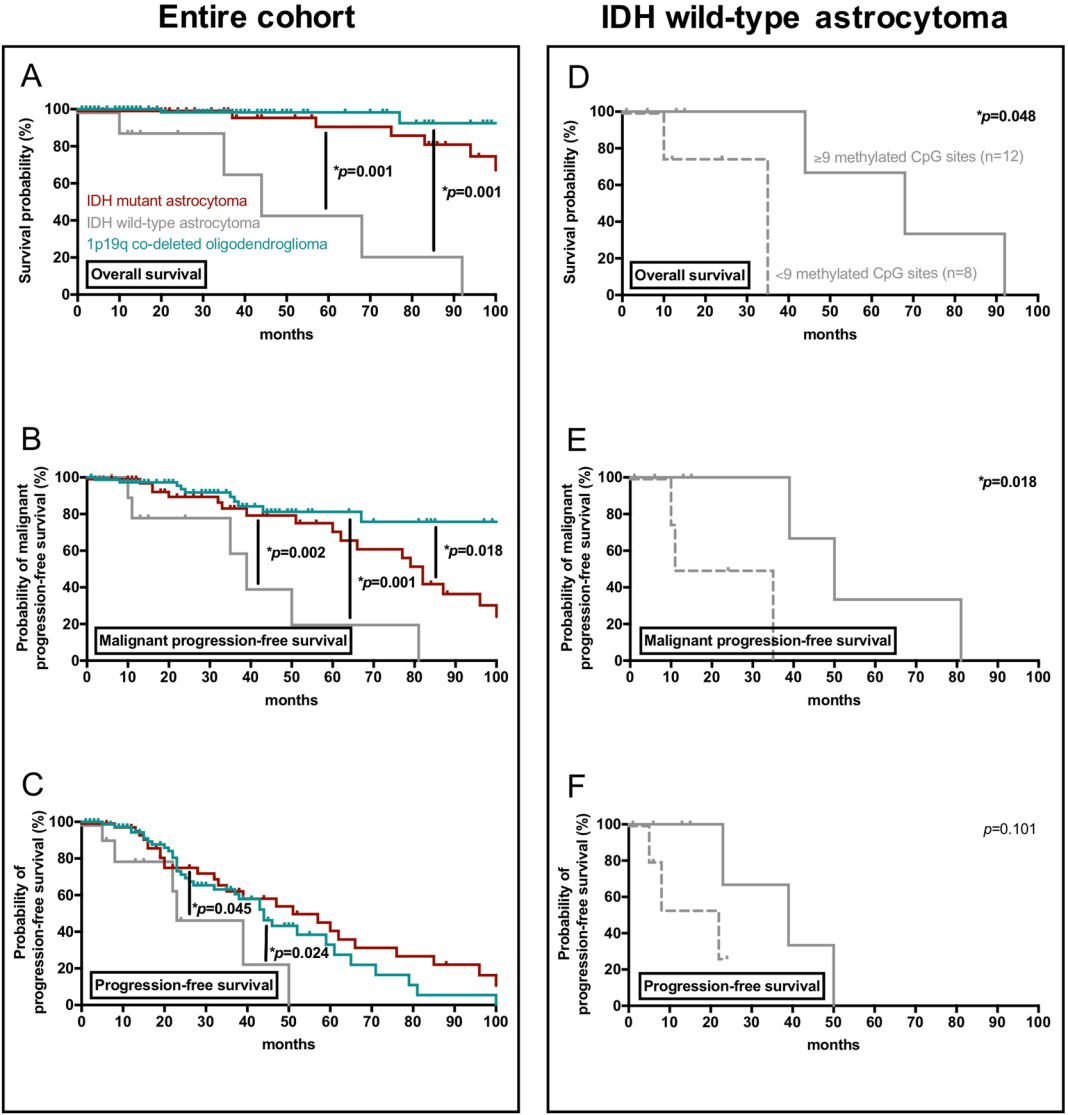
Prognostic markers for survival and disease progression in glioma WHO grade II. (**A**–**C**) Kaplan–Meier estimates of overall survival (**A**), malignant progression-free survival (**B**), and radiographic progression-free survival (**C**) in the entire cohort of glioma WHO grade II. Patients were stratified according to the 2016 WHO classification into 1p19q co-deleted oligodendroglioma (cyan), IDH mutant astrocytoma (red), and IDH wild-type astrocytoma (grey). *p* ≤ 0.05 is displayed. (**D**–**F**) Kaplan–Meier estimates of overall survival (**D**), malignant progression-free survival (**E**), and radiographic progression-free survival (**F**) in IDH mutant astrocytoma. Curves are displayed for patients with ≥ 9 methylated CpG sites (straight lines) and < 9 methylated CpG sites (dotted lines). Tick marks indicate censored patients.

Median follow-up was 35 months (range 3–136 months). 69 patients developed radiographic tumor progression, 34 patients had malignant tumor progression verified by tissue-based analysis, and 15 patients were deceased due to disease progression at database closure. 27 patients were lost to follow-up after a median of 13 months (range 0–133 months). In the entire cohort, median time to radiographic progression was 44 months; median time to malignant progression was 87 months; and median overall survival was not reached after 120 months. Outcome was also calculated after patients were assigned according to three molecular groups based on the 2016 WHO classification (Fig. 2A–C). Patients with astrocytoma, IDH wild-type had significantly shorter time to radiographic and malignant progression as well as lower overall survival when compared to patients with oligodendroglioma, 1p19q co-deleted (radiographic progression: *p* = 0.045; malignant progression: *p* = 0.001; overall survival: *p* = 0.001) and astrocytoma, IDH mutant (radiographic progression: *p* = 0.024; malignant progression: *p* = 0.002; overall survival: *p* = 0.001). Shorter time to malignant progression was also seen in astrocytoma, IDH mutant when compared to oligodendroglioma, 1p19q co-deleted (*p* = 0.018), whereas no differences were seen for overall survival or radiographic progression.

### MGMT promotor methylation and other markers of outcome

A larger number of methylated CpG sites was associated with favourable outcome in the entire cohort of 155 patients with WHO grade II (Table 2). A total of ≥ 5 methylated CpG sites (≥ 5/25 CpG sites, ≥ 20%; n = 151) was a positive prognostic marker for longer time to radiographic progression (*p* = 0.008), time to malignant progression (*p* = 0.001), and improved overall survival (*p* = 0.001) in the entire cohort. Of note, all patients with < 5 methylated CpG sites had astrocytoma, IDH wild-type. Among patients with astrocytoma, IDH wild-type, a total of ≥ 9 methylated CpG sites (≥ 9/25 CpG sites, ≥ 36%; n = 12) was associated with longer time to malignant progression (50 vs. 23 months; *p* = 0.018) and improved overall survival (68 vs. 35 months; *p* = 0.048) (Fig. 2D–F). These findings were also retained when patients with pleomorphic xanthastrocytomas were excluded from outcome analysis. Among patients with oligodendroglioma, 1p19q co-deleted and astrocytoma, IDH mutant, higher numbers of methylated CpG sites were neither prognostic of radiographic progression, malignant progression, nor overall survival. We did not find evidence that methylation of a specific CpG site was associated with a distinct outcome.

**Table 2:** Number of methylated CpG sites within the MGMT promotor region as prognostic factor.

Number of methylated CpG sites (*patients at risk*)	Radiographic progression-free survival			Malignant progression-free survival			Overall survival		
	Hazard ratio	95% confidence interval of HR	*p*-value	Hazard ratio	95% confidence interval of HR	*p*-value	Hazard ratio	95% confidence interval of HR	*p*-value
0 (*3*) vs. ≥ 1 (*152*)	0.15	0.0–4.2	0.262	0.00	0.0–0.3	*0.017	2.76	0.0- >100	0.872
4 (*4*) vs. ≥ 5 (*151*)	0.01	0.0–0.3	*0.008	0.00	0.0–0.0	*0.001	0.00	0.0–0.0	*0.001
5 (*7*) vs. ≥ 6 (*148*)	0.52	0.2–1.7	0.652	0.13	0.0–0.9	*0.041	0.47	0.0–6.6	0.576
6 (*7*) vs. ≥ 7 (*148*)	0.52	0.2–1.7	0.652	0.13	0.0–0.9	*0.041	0.47	0.0–6.6	0.576
7 (*10*) vs. ≥ 8 (*145*)	0.35	0.1–1.3	0.108	0.09	0.0–0.5	*0.009	0.53	0.0–6.8	0.624
8 (*11*) vs. ≥ 9 (*144*)	0.29	0.1–0.9	*0.046	0.06	0.0–0.3	*0.001	0.11	0.0–1.4	0.087
9 (*13*) vs. ≥ 10 (*142*)	0.35	0.1–1.0	*0.040	0.38	0.1–1.2	0.100	0.29	0.1–1.7	0.166
10 (*14*) vs. ≥ 11 (*141*)	0.30	0.1–0.8	*0.016	0.40	0.1–1.3	0.119	0.30	0.1–1.7	0.174
11 (*19*) vs. ≥ 12 (*136*)	0.54	0.2–1.2	0.123	0.83	0.3–2.1	0.702	0.57	0.1–2.5	0.457
12 (*25*) vs. ≥ 13 (*130*)	0.72	0.4–1.4	0.319	1.09	0.5–2.5	0.830	0.62	0.2–2.2	0.463

Univariate analysis for radiographic progression-free, malignant progression-free, and overall survival was performed among glioma WHO grade II patients (n = 155). Number of methylated CpG sites was tested as dichotomous variable. Number of patients at risk is indicated in italic numbers. Hazard ratio, 95% confidence interval of hazard ratio, and *p*-value are given. Asterisks and bold letters indicate *p* ≤ 0.05. *CpG Cytosine-Guanine dinucleotide*, *HR* hazard ratio.

Given that ≥ 5 methylated CpG sites were prognostic of favourable outcome in our entire cohort, we analysed whether patients with ≥ 5 methylated CpG sites may have benefited from use of chemotherapy. In the subgroup of patients with ≥ 5 CpG sites, there was no difference in outcome when radiochemotherapy (n = 19) was compared to radiotherapy (n = 9). Also, patients with ≥ 5 methylated CpG in which chemotherapy in general (alone or in combination with radiotherapy; n = 67) was provided did not have more favourable outcome when compared to patients which were treated with approaches other than chemotherapy (microsurgical tumor resection, radiotherapy, interstitial brachytherapy, wait-and-scan; n = 84) on univariate analysis. This also held true when tested only in astrocytoma, IDH wild-type with ≥ 9 methylated CpG sites. Of note, neither chemotherapy nor radiochemotherapy was a predictive marker of outcome among all patients with astrocytoma, IDH wild-type.

In the entire cohort, loss of ATRX expression (n = 46) was associated with shorter time to malignant progression (*p* = 0.001), but not with time to radiographic progression (*p* = 0.819) or overall survival (*p* = 0.809). When stratified into the three groups, loss of ATRX expression (n = 44) retained its role as a negative prognostic marker for malignant progression only among astrocytoma, IDH mutant (*p* = 0.029). TERT mutation was not associated with outcome among patients with astrocytoma, IDH wild-type.

## Discussion

MGMT promotor methylation is considered an important biomarker for outcome in glioma WHO grade III and IV. The association of MGMT promotor methylation and favourable outcome is less well established in glioma WHO grade II. Only two prospective clinical trials (RTOG 0424 and EORTC 22033–26033) have reported on MGMT promotor status among patients with glioma WHO grade II, and both trials have produced inconclusive evidence whether MGMT promotor methylation is independent from other molecular markers^5,6^. Based on a large cohort of 155 patients, we here present the institutional experience for the role of MGMT promotor methylation in glioma WHO grade II.

We found that extent of MGMT promotor methylation was highest when both IDH mutation and 1p19q co-deletion were present, and exceedingly lower as well as more variable when both molecular markers were absent. Our findings are supported by data from the RTOG 0424 trial, which almost exclusively encountered astrocytoma, IDH wild-type among glioma WHO grade II with an unmethylated MGMT promotor status^6^. In turn, the presence of both 1p19q co-deletion and IDH mutation was strictly linked to MGMT promotor methylation in the EORTC 22033–26033 trial^5^. However, MGMT promotor status was reported in a rather binary fashion as either 'methylated' or 'unmethylated' in these trials. In contrast, Sanger sequencing of 25 individual CpG sites allowed the exact quantification of the number of methylated CpG sites in the present study. Using this approach, we were able to show that also significant differences in extent of methylation among IDH mutated tumors with or without 1p19q co-deletion exist. Our findings corroborate the hypothesis that there may be a nested dependency of MGMT promotor methylation, IDH mutation, and 1p19q co-deletion.

In our entire cohort, a higher number of methylated CpG sites represented a positive prognostic factor for improved overall survival and longer time to tumor progression. These observations are in line with previous retrospective analyses including an evaluation of data from the RTOG 0424 trial^6,13^. Molecular markers will increasingly be incorporated into the classification of glioma WHO grade II according to the recent cIMPACT-NOW update, and we therefore further stratified our patients based on their molecular signature^2^.

Surprisingly, our analysis showed that the prognostic value of MGMT promotor methylation was only retained in patients with astrocytoma, IDH wild-type, but not in IDH mutant glioma with or without 1p19q co-deletion. This might be due to the fact that the presence of IDH mutation may represent a more dominant prognostic factor than MGMT promotor methylation as it has been previously suggested^14,15^. Of note, astrocytic tumors without IDH mutation previously graded as WHO grade II were suggested to be denoted as glioblastoma WHO grade IV in the presence of either a TERT mutation, EGFR amplification, or + 7/− 10 chromosome copy number changes according to the recent cIMPACT-NOW update^2^. Only TERT mutation status was available for our review and was not prognostic in our cohort, potentially due to our limited sample size. We cannot rule out that a number of astrocytic tumors without IDH mutations may had EGFR amplification or chromosome copy number changes. Given that these data were not available in our study, we denoted all of our astrocytic tumors without IDH mutation as WHO grade II according to the 2016 WHO classification. Future studies may perform a more thorough molecular characterization and potentially re-classify such tumors as WHO grade IV according to the most recent cIMPACT-NOW update^2^. Our study seems to support the hypothesis that MGMT promotor status may not only add prognostic information in histologically defined glioblastoma, but also in astrocytic tumors without IDH mutation. Our prognostic cut-off points appear consistent with what has been reported in glioma WHO grade III and IV; although comparison is hampered across various institutions as different methods on determining MGMT promotor status are being used^16,17^.

A definitive consensus on methods and cut-off for defining MGMT promotor methylation remains to be found. The most popular techniques in clinical use include methylation-specific PCR and pyrosequencing, but other approaches including genome-wide analyses are also available. Although most techniques have been prospectively validated to identify patients with and without MGMT promotor methylation^18^, the methods dramatically differ in regard of reproducibility, inter-observer variability, and predictive value for outcome^19^. Pyrosequencing of CpG sites within the MGMT promotor region might be the most reliable and easy-to-use method which has emerged for routine testing^20^; however, most laboratories using sequencing only analyse 4 or less out of 98 CpG sites within the MGMT promotor^21^. All methods including pyrosequencing characteristically report MGMT promotor methylation as present or absent, which may jeopardize cases with borderline methylation which have been previously reported^18,22^. In contrast, our approach using Sanger sequencing allowed an objective and quantitative rather than a binary analysis of the MGMT promotor region. The quantitative data can be used to objectively determine prognostic cut-offs for MGMT promotor methylation within different subgroups, and to highlight differences among tumors which would otherwise be just characterized as 'methylated' or 'unmethylated'. On a cautionary note, such a method is rather time-consuming as CpG site methylation needs to be determined separately for each of the numerous CpG sites.

Importantly, our findings on the prognostic role of MGMT promotor methylation will need to be interpreted with high caution as patients in our cohort underwent heterogeneous therapeutic approaches. Sample sizes of treatment-based subgroups were rather small in our study, which might have confounded our analysis. This also limits interpretation of the prognostic role of ATRX and TERT. Moreover, future studies on the relevance of MGMT promotor methylation will need to discuss whether patients with pleomorphic xanthastrocytomas should be excluded from the larger group of gliomas WHO grade II given that such patients may have distinct outcome compared to diffuse glioma^1^, and the relevance of MGMT promotor status is highly unclear in this peculiar cohort^23^. Implications of MGMT promotor status and other molecular markers on outcome warrants further evaluation in prospective cohorts of glioma WHO grade II which have been treated with uniform therapeutic approaches.

MGMT promotor methylation may predict response to chemotherapy in glioma WHO grade III and IV^24,25^. However, we were not able to detect improved outcome for patients with higher numbers of methylated CpG sites which were treated with chemotherapy when compared to other therapeutic approaches. This was particularly also true for astrocytoma, IDH wild-type. Given that none of our patients with a low number of methylated CpG sites received chemotherapy or radiochemotherapy, however, we cannot comment on whether higher numbers of methylated CpG sites may indeed indicate increased chemosensitivity. The RTOG 0424 trial and the EORTC 22033–26033 trial have both also produced inconclusive data in this regard^5,6,26^. It therefore remains to be shown in future studies whether MGMT promotor methylation indicates that the use of chemotherapy translates into improved outcome in glioma WHO grade II.

Collectively, extent of MGMT promotor methylation in glioma WHO grade II depends on IDH mutation and on 1p19q co-deletion. Quantification of MGMT promoter methylation may add prognostic information in patients with astrocytoma, IDH wild-type. Implications of MGMT promotor methylation on outcome in glioma WHO grade II warrants evaluation in prospective clinical cohorts undergoing standardized therapeutic approaches. Understanding the biological role of MGMT promotor status in glioma WHO grade II may be useful in future management of such patients.

## Data Availability

All relevant data are within the paper. All data were kept anonymous and are available on qualified request.
